# Application of intra-arterial chemotherapy in high-risk non-muscle invasive bladder cancer: a systematic review and meta-analysis

**DOI:** 10.7717/peerj.12248

**Published:** 2021-09-28

**Authors:** Chengyu You, Xianhui Li, Yuelin Du, Hui Wang, Xiaojun Zhang, Tangqiang Wei, Anguo Wang

**Affiliations:** Nanchong Central Hospital, The Second Clinical College, North Sichuan Medical College, Nanchong, China

**Keywords:** Bladder cancer, Intra-arterial chemotherapy, Intravesical chemotherapy, Recurrence, Survival

## Abstract

**Background:**

To summarize the current evidence on the effects of intra-arterial chemotherapy (IAC) on high-risk non-muscle invasive bladder cancer (NMIBC) and compare oncology results with intravesical chemotherapy (IVC).

**Methods:**

We performed a systematic review and cumulative meta-analysis of the primary outcomes of interest by a systematical search of multiple scientific databases in February 2021. The mean difference (MD) and odds ratio (OR) were calculated for continuous and dichotomous variables respectively, with 95% confidence intervals (CIs). The hazard radio (HR) with 95% CIs was used for overall survival (OS), recurrence-free survival (RFS) and progression-free survival (PFS).

**Results:**

A total of six studies with 866 patients were included. For IAC combined with IVC versus IVC alone, statistically significant differences were found regarding tumor recurrence rate (OR: 0.51, 95% CI [0.36∼0.72], *p* = 0.0001), tumor progression rate (OR: 0.47, 95% CI [0.30∼0.72], *p* = 0.0006), tumor-specific death rate (OR: 0.49, 95% CI [0.25∼0.99], *p* = 0.05), PFS (HR: 0.47, 95% CI [0.23∼0.96], *p* = 0.04) and RFS (HR: 0.60, 95% CI [0.41∼0.87], *p* = 0.007). No significant difference between two groups was found for time to first recurrence (MD: 3.27, 95% CI [−2.37∼8.92], *p* = 0.26) and OS (HR: 1.20, 95% CI [0.44∼3.32], *p* = 0.72). For IAC alone versus IVC, There was no statistical difference in the terms of tumor-specific death rate (OR: 0.67, 95% CI [0.29∼1.53], *p* = 0.34), RFS (HR: 0.90, 95% CI [0.56∼1.46], *p* = 0.68) and PFS (HR: 0.71, 95% CI [0.32∼1.55], *p* = 0.39). Adverse events mainly included nausea/vomiting (36.3%), hypoleukemia (19.4%), neutropenia (16.0%), increased creatinine (9.9%), increased alanine aminotransferase (18.7%), and thrombocytopenia (9.9%).

**Conclusion:**

The IAC combined with IVC is a safe and effective treatment for high risk NMIBC, with lower rates of recurrence, progression, tumor-specific death, PFS and RFS, and with minor and tolerable events. The effectiveness of the IAC alone is parallel to the IVC alone.

## Introduction

Bladder cancer (BC) is the 7th most common cancer in the male population worldwide, and its incidence is about four times greater than in women (Bray et al., 2018; Ferlay et al., 2019). The non-muscle invasive BC (NMIBC) accounts for about 75%. Approximately 25% of NIMBC are high-risk with poor prognosis, whose recurrence and progression range from 62 to 78% and 17 to 45% at 5 years, respectively (Humphrey et al., 2016; Sylvester et al., 2010).

At present, both bladder-preserving therapy and cystectomy are commended for high-risk NMIBC (Babjuk et al., 2020). However, the cystectomy considered an excessive treatment and decreased the quality of life. On the other hand, the adjuvant intravesical therapy remains highly controversial because of its prognosis (De Berardinis et al., 2011; Thalmann et al., 2004). Therefore, intra-arterial chemotherapy (IAC) is discussed widely. It was administered by a modified Seldinger technique, which placed the percutaneous catheter system in the bilateral internal iliac arteries, and an angiographic catheter was passed to the targeted artery. The end of the catheter was connected buried underneath the skin, and then regularly injected chemotherapy drug (Huang et al., 2019b). Previous studies have suggested that IAC reduced the recurrence and progression of NIMBC comparing with intravesical chemotherapy (IVC) (Eapen et al., 2004). The current meta-analysis only showed the IAC combined with IVC and ignored the IAC alone for NMIBC (Zhou et al., 2021). What’s worse, it included two studies that overlap, which reduced its quality of the evidence. Hence, we performed a systematic review and meta-analysis to discuss both IAC combined with IVC and IAC alone for patients with high-risk NMIBC.

## Materials & Methods

This review was performed according to the Preferred Reporting Items for Systematic Reviews and Meta-analysis (PRISMA) Statement (Shamseer et al., 2015). The methods of it were registered prospectively (CRD42020220512) in the PROSPERO.

### Literature search

A systematic literature search was performed in February 2021 using PubMed and the Cochrane Library databases. Search terms included bladder cancer, intra-arterial chemotherapy, and intravesical chemotherapy. No restrictions were put on publication language and date. In addition, we also manually retrieved references which come from relevant studies. The detailed search formula was presented in supplementary material.

The studies involved patients with high-risk NMIBC who underwent bladder-preserving operation were included, which compared IAC combined with IVC and IVC alone, or compared IAC and IVC alone. Besides, we did not include letters, cases, reviews, conference abstracts and studies which are irrelevant to the theme or lack complete data.

### Data extraction

All outcomes of interest were collected in a piloted form including the author, publication year, study design, participant characteristics (age, follow-up, chemotherapy methods, and chemotherapy drugs), tumor recurrence rate, time to first recurrence, tumor progression rate, tumor-specific death rate, overall survival (OS), recurrence-free survival (RFS), progression-free survival (PFS), and IAC related adverse events. The Engauge Digitizer version 4.1 (http://digitizer.sourceforge.net/) was used to excavate data from Kaplan Meier-curve for the included studies. And all adverse events were recorded, defined, and graded according to National Cancer Institute common toxicity criteria for adverse events (CTCAE).

### Quality assessment

The quality of randomized controlled trial (RCT) and retrospective studies were evaluated by the Jadad scale and Newcastle–Ottawa scale (NOS), respectively (Clark et al., 1999; Rucker et al., 2021). The total score of the Jadad ≥ 4 or the NOS ≥ 7 is considered as high quality. Moreover, the level of evidence of each study was assessed according to the Oxford Centre for Evidence-Based Medicine 2011 Levels of Evidence (Guyatt et al., 2008). Meanwhile, the risk of bias was independently assessed using the standard Cochrane Collaboration risk-of-bias tool for single-arm studies and the Risk of Bias in Non-Randomized Studies–of Interventions tool (ROBINS-I) for comparative studies (Higgins et al., 2011; Sterne et al., 2016).

The above steps were completed by two of us (CY.Y and H.W) independently. After discussion, the disagreements were resolved by the senior author (AG.W).

### Statistical analysis

For continuous and dichotomous variables, the mean difference (MD) and odds ratio (OR) were applied respectively, with their 95% confidence intervals (CIs). For adverse events, the statistical formulas were used to convert the effect indicators, including P = OR/(1+OR), the low limit (LL) = LL_OR_/(1+LL_OR_), and the upper limit (UL) = UL_OR_/(1+UL_OR_). For terms of OS, RFS and PFS, the hazard radio (HR) with its 95% CIs were employed. The chi-squared and I- squared test were considered to assess the heterogeneity of included studies. Fixed-effects models were used for low heterogeneity (I^2^ < 90%). On the contrary, the random-effects models were used for high heterogeneity (I^2^ > 90%). Finally, *P* value of <0.05 was taken as a statistically significant index. All statistical analysis were completed by the Review Manager software (RevMan) Version 5.3.

## Results

**Figure 1:**
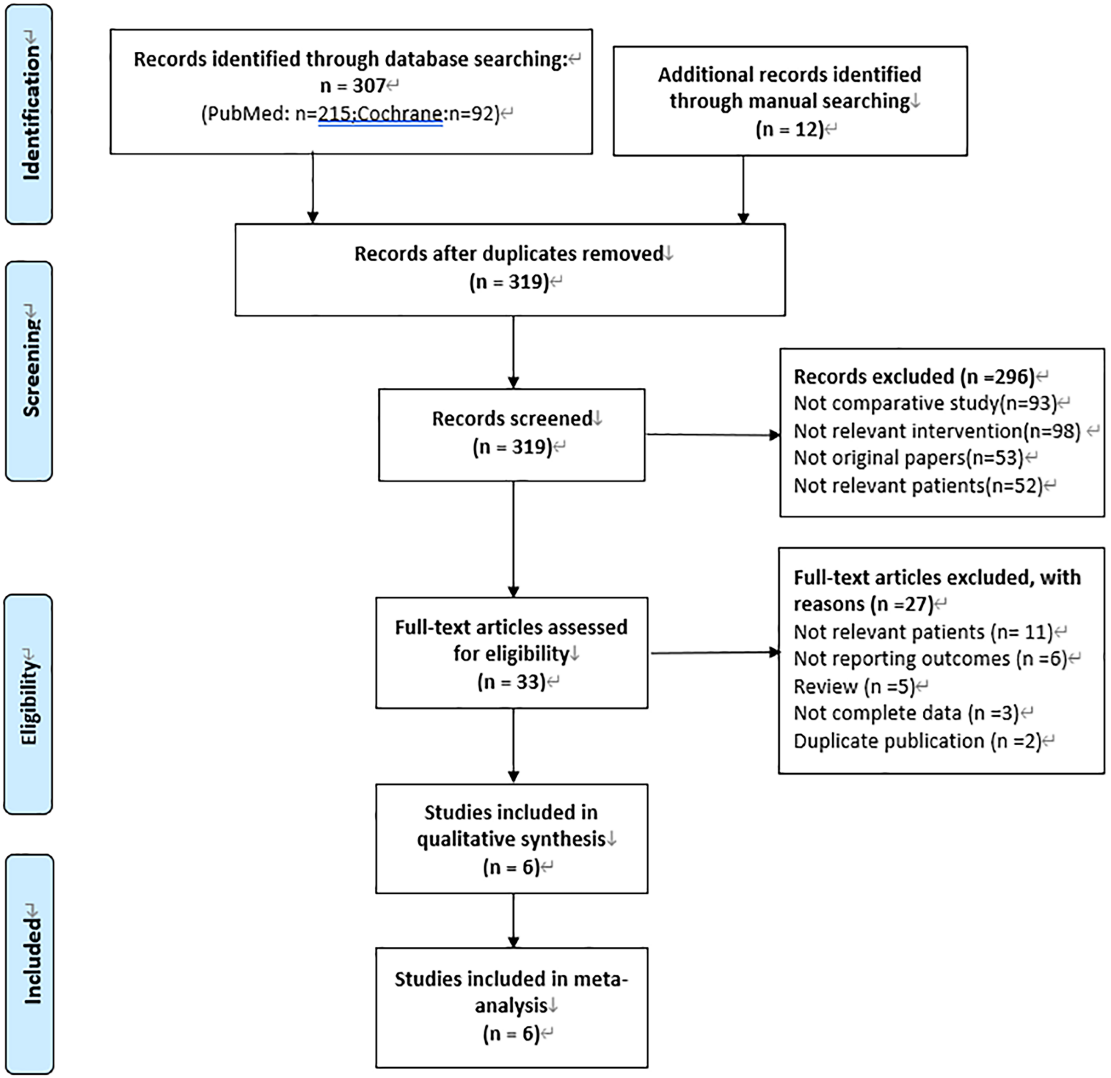
PRISMA flow diagram.

**Figure 2:**
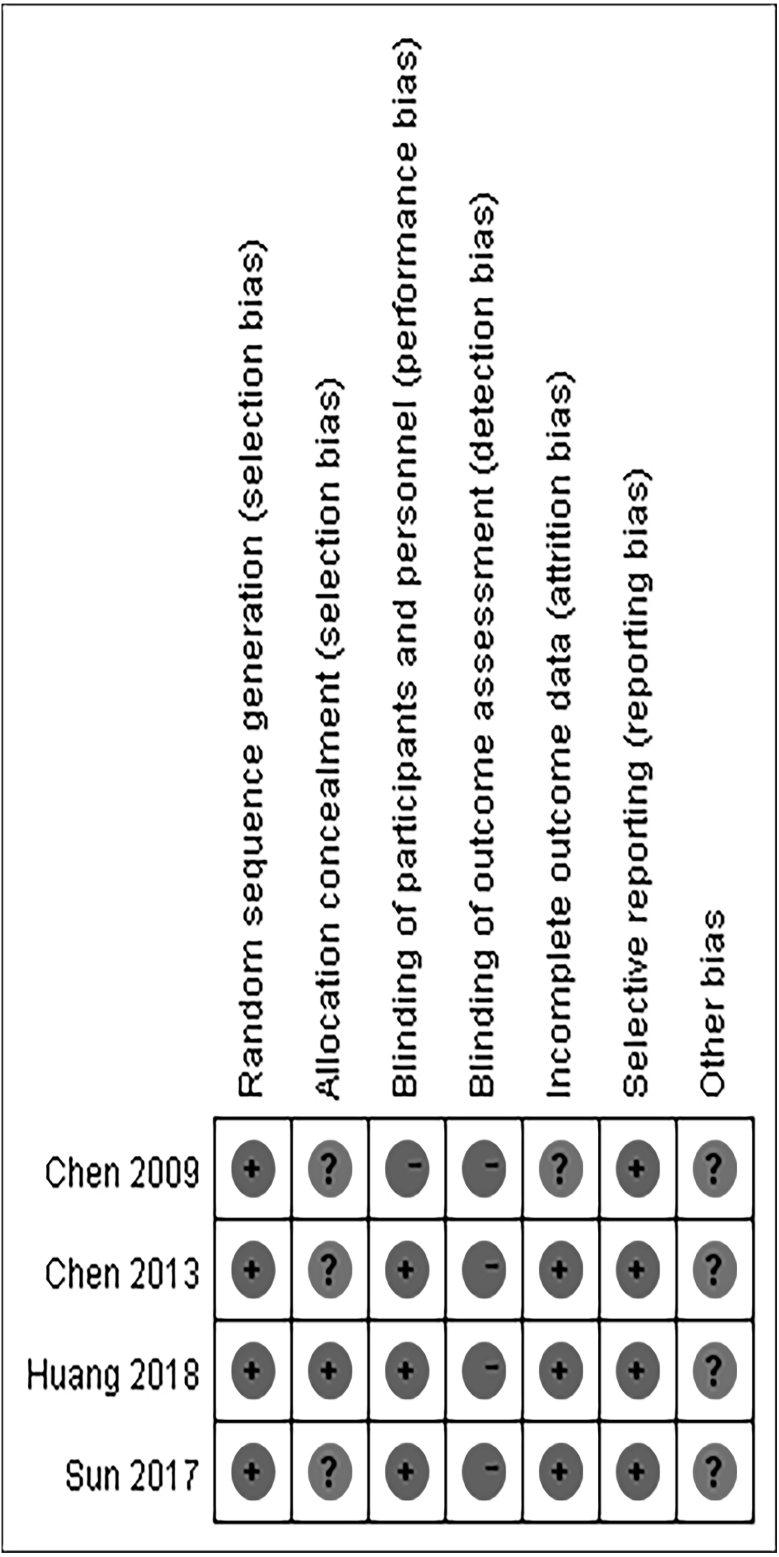
Risk of bias for included studies.

After literature search, 319 studies were identified. A total of 6 studies (Chen et al., 2013; Chen et al., 2009; Huang et al., 2019a; Lian et al., 2019; Liu et al., 2018; Sun et al., 2017) were included in our meta-analysis in the end by excluding duplication, irrelevant records and low-quality studies. Among them, 4 studies (Chen et al., 2013; Huang et al., 2019a; Lian et al., 2019; Sun et al., 2017) compared IAC combined with IVC and IVC alone, and others (Chen et al., 2009; Liu et al., 2018) compared IAC and IVC. The specific process is shown in Fig. 1. The characteristics of included studies and their quality scores are shown in Table 1. A high risk of bias was recorded for the 4 RCTs (Chen et al., 2013; Chen et al., 2009; Huang et al., 2019a; Sun et al., 2017), and whilst a moderate risk of bias was recorded for the remaining 2 retrospective studies (Lian et al., 2019; Liu et al., 2018) (Fig. 2).

### IAC combined with IVC *versus* IVC alone

**Figure 3:**
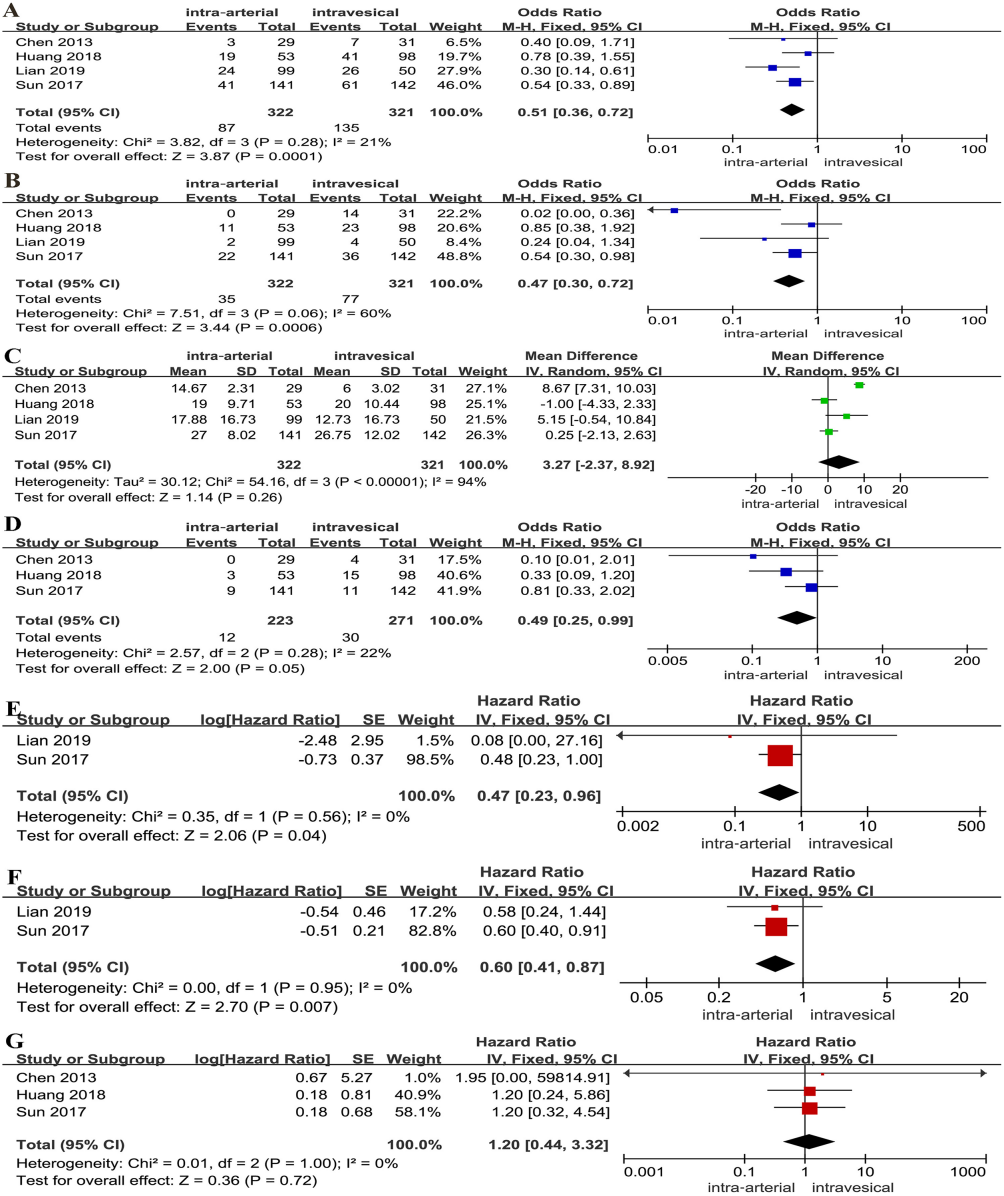
Forest plot and meta-analysis of tumor recurrence rate (A), tumor progression rate (B), time to first recurrence (C), tumor-specific death rate (D), PFS (E), RFS (F) and OS (G) for IAC combined with IVC *versus* IVC alone.

A lower tumor recurrence rate (OR: 0.51, 95% CI [0.36∼0.72], *p* = 0.0001, Fig. 3A) and tumor progression rate (OR: 0.47, 95% CI [0.30∼0.72], *p* = 0.0006, Fig. 3B) were associated with IAC combined with IVC. However, no significant differences between two groups were found for time to first recurrence (MD: 3.27, 95% CI [−2.37∼8.92], *p* = 0.26, Fig. 3C). A lower tumor-specific death rate was relevant with IAC combined with IVC (OR: 0.49, 95% CI [0.25∼0.99], *p* = 0.05, Fig. 3D). For survival outcomes, a statistically significant difference was found regarding PFS (HR: 0.47, 95% CI [0.23∼0.96], *p* = 0.04, Fig. 3E) and RFS (HR: 0.60, 95% CI [0.41∼0.87], *p* = 0.007, Fig. 3F). Yet, there was no clinically meaningful differences for OS (HR: 1.20, 95% CI [0.44∼3.32], *p* = 0.72, Fig. 3G).

**Table 1:** Characteristics and quality assessment of included studies.

Study	Study design	NO. of patients	Age (years)	Chemotherapy methods	Chemotherapy drugs (IAC/IC)	Follow-up	Quality^c^	Level of evidence^d^
Chen2009	RCT	25/27	57(31–82)/60(34–87)^a^	IAC/IC	Gemcitabine + cisplatin/epirubicin	40(6–67)/42(6–67)^a^	4	1B
Chen2013	RCT	29/31	63(30–80)/65(29–83)	IAC+IC/IC	Epirubicin + cisplatin/epirubicin	22(5–58)/23(11–58)^a^	4	1B
Huang2018	RCT	53/98	68(30–84)/67(29–82)^a^	IAC+IC/IC	pirarubicin + cisplatin/pirarubicin	79(7–131)/59(7–127) ^a^	6	1B
Lian2019	R	99/50	60.65 ± 12.64/63.3 ± 12.79^b^	IAC+IC/IC	Epirubicin + cisplatin/epirubicin	24.25(5–50)/22.3(10–42)^a^	7	4
Liu2018	R	62/141	59.6 ± 11.6/62.9 ± 11.2^b^	IAC/IC	Gemcitabine + cisplatin/epirubicin	57.5 ± 42.3/48.3 ± 35.9^b^	8	4
Sun2017	RCT	141/142	69.59 ± 11.02/69.03 ± 11.01^b^	IAC+IC/IC	Epirubicin + cisplatin/epirubicin	47.3(16–78)/46.8(13–076)^b^	5	1B

**Notes.**

RCT randomized controlled trial R retrospective study IAC Intra-arterial chemotherapy IC Intravesical chemotherapy

a Median (range).

b Mean ± SD.

c Using the Jadad or NOS scale.

d According to the Oxford Centre for evidence-based medicine 2011 levels of evidence.

### IAC alone *versus* IVC alone

**Figure 4:**
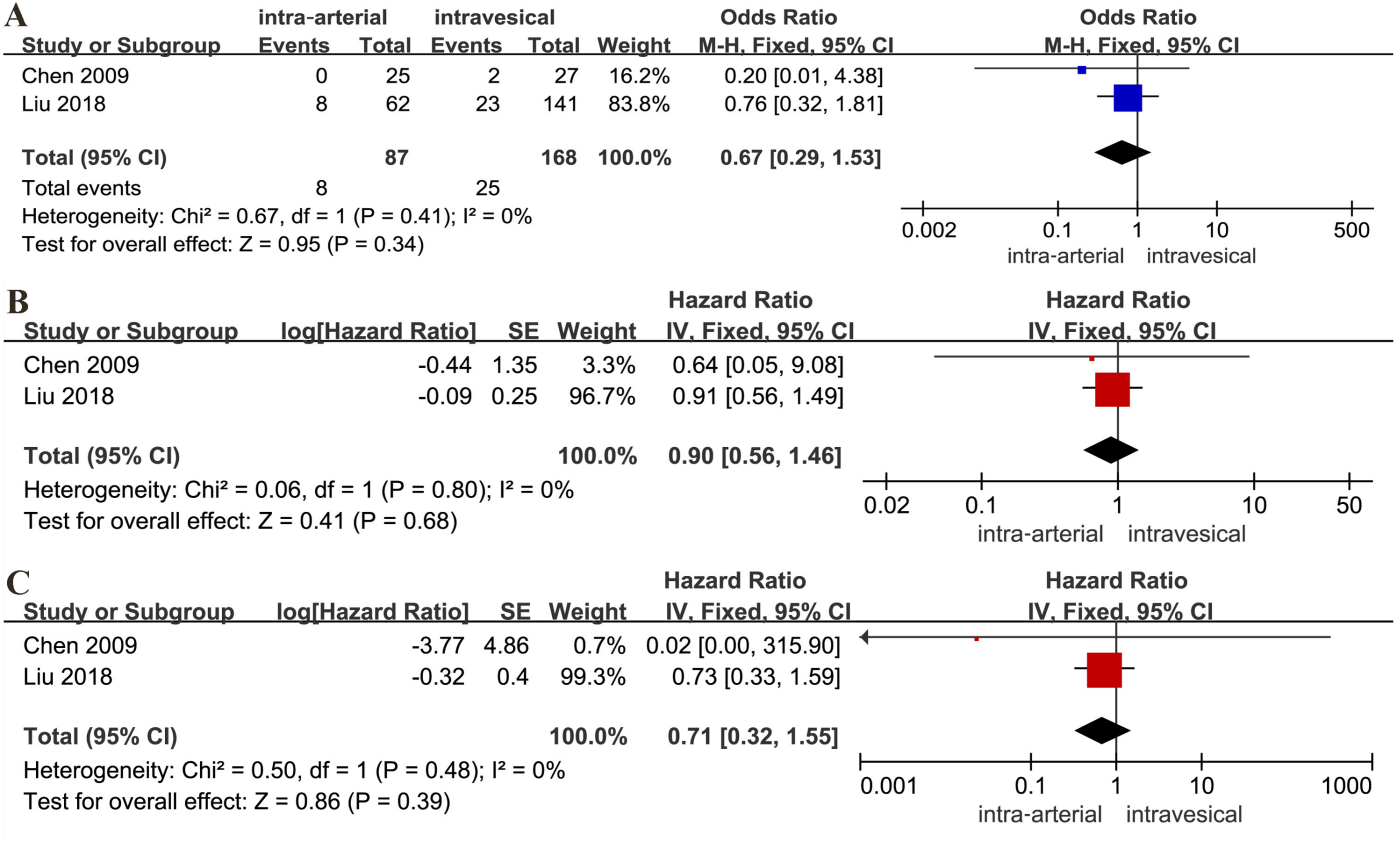
Forest plot and meta-analysis of tumor-specific death rate (A), RFS (B) and PFS (C) for IAC alone *versus* IVC alone.

There was no statistical difference between the IAC and IVC for tumor-specific death rate (OR: 0.67, 95% CI [0.29∼1.53], *p* = 0.34, Fig. 4A), RFS (HR: 0.90, 95% CI [0.56∼1.46], *p* = 0.68, Fig. 4B) and PFS (HR: 0.71, 95% CI [0.32∼1.55], *p* = 0.39, Fig. 4C).

### Adverse events

The main adverse events of IAC included nausea/vomiting (OR: 0.363, 95% CI [0.259∼0.476], *p* = 0.02), hypoleukemia (OR: 0.194, 95% CI [0.099∼0.359], *p* = 0.0008), neutropenia (OR: 0.160, 95% CI [0.083∼0.286], *p* < 0.0001), increased creatinine (OR: 0.099, 95% CI [0.065∼0.160], *p* < 0.00001), increased alanine aminotransferase (OR: 0.187, 95% CI [0.130∼0.265], *p* < 0.00001), and thrombocytopenia(OR: 0.099, 95% CI [0.029∼0.275], *p* = 0.0004) (Table 2).

**Table 2:** Meta-analysis of adverse events.

Adverse events	Number of included studies	Heterogeneity (I^2^)	OR (95% CI)	*P*
nausea/vomiting	4	59%	0.363 (0.259,0.476)	0.02
hypoleukemia	4	75%	0.194 (0.099,0.359)	0.0008
neutropenia	4	78%	0.160 (0.083,0.286)	<0.0001
increased creatinine	4	0%	0.099 (0.065, 0.160)	<0.00001
increased alanine aminotransferase	3	0%	0.187 (0.130,0.265)	<0.00001
thrombocytopenia	3	83%	0.099 (0.029,0.275)	0.0004

**Notes.**

I^2^ I- squared test OR odds ratio 95% CI 95% confidence intervals (CIs)

## Discussion

At present, IVC with Bacille Calmette–Guerin (BCG) is widely accepted (Babjuk et al., 2020). However, fewer than half completed the cycle of full-dose BCG treatment because of adverse complications (Kulkarni et al., 2007). Subsequently, various treatments were explored, such as new drugs, radiation therapy, and novel chemotherapy approaches including intravenous chemotherapy, IAC and hyperthermic intravesical chemotherapy (Chiancone et al., 2020; Di Lorenzo et al., 2010; Weiss et al., 2006; Yang et al., 2017). The IAC had received attention because of higher chemotherapy concentration in the bladder and lower systemic toxicity compared with intravenous chemotherapy (Eapen et al., 2004).

On one hand, our results suggested that IAC combined with IVC was superior to IVC alone in terms of recurrence, progression, tumor-specific death rate, PFS and RFS, because of a higher concentration and a better distribution of anti-tumor drugs in the tumor organ. The former attacked the bladder mucosal layer by IVC, and infiltrated the bladder cancer from the blood supply by IAC (Hoshi et al., 1997; Huang et al., 2019a). However, there is no significant differences between two groups for time to first recurrence with high heterogeneity. The presence or absence of carcinoma *in situ* (CIS) plays main roles in the high heterogeneity. Because previous studies had reported that CIS involvement predicts poor prognosis, and is related to obviously higher incidence of progression (Solsona et al., 1996; Sylvester et al., 2005). Huang et al. reported that a shorter time to first recurrence is associated with the IAC combined with IVC by excluding patients with CIS (*p* = 0.028), which proved that the IAC combined with IVC has a great potential to extend recurrence time (Huang et al., 2019a). In addition, Chen et al. (2013) found that the tumor mainly deteriorated in the first 20 months and that later recurrences were rare. Therefore, we speculated that the timing, frequency and order of the two chemotherapy methods in the short term may influence its recurrence and progression, just as the timing of IVC (Perlis et al., 2013). Lian et al. (2019) thought that the number of tumors and pathological stage were closely related to recurrence. Besides, previous studies had proved that drugs are concerned with recurrence, which contribute to high heterogeneity (Malmstrom et al., 2009; Yang et al., 2017). By the way, Chiancone et al recently reported that systemic inflammatory markers were significantly associated with bladder cancer recurrence or progression, including C-reactive protein, erythrocyte sedimentation rate and neutrophil-to-lymphocyte ratio (Chiancone et al., 2021). Due to the underlying diseases and accidents, the results of OS have no clinical significance.

On the other hand, although included studies both found that a statistically significant differences were found in the long-term oncology outcomes, out results suggested that there were no significant differences between the IAC and IVC alone. Obviously, it was no clinical significance because of small sample size, the potential of performance, detection and publication biases, which needs further quality studies to verify.

For adverse events, the nausea/vomiting was most common events (36.3%). Chen et al. found that most toxicities were minor and reversible without intervention (46.7% *versus* 6.9%), which is consistent with previous studies (Chen et al., 2013; Chen et al., 2009; Eapen et al., 2004). Furthermore, no severe arterial complications and no patients who discontinued treatment due to adverse reactions were reported in all included studies. Lian et al. thought that learning curve and initial experience were associated with a better safety profile and tolerability (Lian et al., 2019).

There were still some limitations. First, due to contingency, potential publication bias and selection bias, the quality of evidence was low. Second, some data are not suitable for merging because of insufficient follow-up. Finally, computer-based cannot collect all relevant research, which was one source of publication bias.

## Conclusion

In conclusion, our systematic review and meta-analysis indicates that the IAC combined with IVC is a safe and effective treatment for high-risk NMIBC, with lower rates of recurrence, progression, tumor-specific death, PFS and RFS, and with minor and tolerable events. The effectiveness of the IAC alone is parallel to the IVC alone. However, further quality studies are needed to evaluate its effectiveness due to low quality of evidence.

## Supplemental Information

10.7717/peerj.12248/supp-1 Supplemental Information 1 PRISMA checklist

10.7717/peerj.12248/supp-2 Supplemental Information 2 Rationale

10.7717/peerj.12248/supp-3 Supplemental Information 3 The detailed search formula

## Additional Information and Declarations

### Data Availability

The following information was supplied regarding data availability: The raw measurements are available in the figures and tables.
