# Supplementary material for: Application of intra-arterial chemotherapy in high-risk non-muscle invasive bladder cancer: a systematic review and meta-analysis
Source: PeerJ. 2021 Sep 28;9:e12248. doi: 10.7717/peerj.12248 (PMC8485834; doi:10.7717/peerj.12248)
Supplement: Supplemental Information 1 [file peerj-09-12248-s001.doc]

| **Section/topic** | **#** | **Checklist item** | **Reported on page #** |
| --- | --- | --- | --- |
| **TITLE** | | |  |
| Title | 1 | Identify the report as a systematic review and meta-analysis. | 1 |
| **ABSTRACT** | | |  |
| Structured summary | 2 | The structured abstract includes Background, Methods, Results, Conclusion. | 1 |
| **INTRODUCTION** | | |  |
| Rationale | 3 | Described in the Introduction. | 2 |
| Objectives | 4 | Stated in the Introduction. | 2 |
| **METHODS** | | |  |
| Protocol and registration | 5 | Both provided in the Materials & Methods. | 2 |
| Eligibility criteria | 6 | Provided in the Materials & Methods. | 2 |
| Information sources | 7 | Provided in the Materials & Methods. | 2 |
| Search | 8 | Provided in the Materials & Methods. | 2 |
| Study selection | 9 | Provided in the Fig1. | 2 |
| Data collection process | 10 | Provided in the Materials & Methods. | 3 |
| Data items | 11 | Provided in the Materials & Methods. | 3 |
| Risk of bias in individual studies | 12 | Provided in the Materials & Methods. | 3 |
| Summary measures | 13 | Provided in the Materials & Methods. | 3 |
| Synthesis of results | 14 | Provided in the Materials & Methods. | 3 |

Page 1 of 2

| **Section/topic** | **#** | **Checklist item** | **Reported on page #** |
| --- | --- | --- | --- |
| Risk of bias across studies | 15 | Provided in the Materials & Methods. | 3 |
| Additional analyses | 16 | Provided in the Materials & Methods. | 3 |
| **RESULTS** | | |  |
| Study selection | 17 | Provided in the Results. | 3 |
| Study characteristics | 18 | Provided in the Table 1. | 4 |
| Risk of bias within studies | 19 | Provided in the Fig2. | 4 |
| Results of individual studies | 20 | Provided in the Results. | 4 |
| Synthesis of results | 21 | Provided in the Results. | 4 |
| Risk of bias across studies | 22 | Provided in the Results. | 4 |
| Additional analysis | 23 | Additional analysis does not apply. |  |
| **DISCUSSION** | | |  |
| Summary of evidence | 24 | Not applicable. |  |
| Limitations | 25 | Discuss limitations at study and outcome level (e.g., risk of bias), and at review-level (e.g., incomplete retrieval of identified research, reporting bias). | 6 |
| Conclusions | 26 | Provide a general interpretation of the results in the context of other evidence, and implications for future research. | 6 |
| **FUNDING** | | |  |
| Funding | 27 | Describe sources of funding for the systematic review and other support (e.g., supply of data); role of funders for the systematic review. |  |

*From:*  Moher D, Liberati A, Tetzlaff J, Altman DG, The PRISMA Group (2009). Preferred Reporting Items for Systematic Reviews and Meta-Analyses: The PRISMA Statement. PLoS Med 6(6): e1000097. doi:10.1371/journal.pmed1000097

For more information, visit: **www.prisma-statement.org**.

Page 2 of 2
