# Supplementary material for: Application of intra-arterial chemotherapy in high-risk non-muscle invasive bladder cancer: a systematic review and meta-analysis
Source: PeerJ. 2021 Sep 28;9:e12248. doi: 10.7717/peerj.12248 (PMC8485834; doi:10.7717/peerj.12248)
Supplement: Supplemental Information 2 [file peerj-09-12248-s002.docx]

**The rationale for conducting the systematic review/meta-analysis.**

Bladder cancer (BC) is the 7th most common cancer in the male population worldwide, and its incidence is about four times greater than in women. The non-muscle invasive BC (NMIBC) accounts for about 75%. Approximately 25% of NIMBC are high-risk with poor prognosis, whose recurrence and progression range from 62 to 78% and 17 to 45% at 5 years, respectively.

At present, both bladder-preserving therapy and cystectomy are commended for high‑risk NMIBC. However, the cystectomy considered an excessive treatment and decreased the quality of life. On the other hand, the adjuvant intravesical therapy remains highly controversial because of its prognosis. Therefore, intra-arterial chemotherapy (IAC) is discussed widely. What’s more, some previous studies have suggested that IAC reduced the recurrence and progression of NIMBC comparing with intravesical chemotherapy (IVC). Hence, we performed a systematic review and meta-analysis to discuss the effectiveness of intra-arterial chemotherapy in patients with high‑risk NMIBC.

**The contribution that the it makes to knowledge in light of previously published related reports, including other meta-analyses and systematic reviews.**

At present, IVC with Bacille Calmette–Guerin(BCG) is widely accepted for high‑risk NMIBC. However, fewer than half completed the cycle of full-dose BCG treatment because of adverse complications. The IAC had received attention because of higher chemotherapy concentration in the bladder and lower systemic toxicity compared with intravenous chemotherapy.

In our study, a total of 6 studies with 866 patients were included. Our systematic review and meta-analysis indicates that the IAC combined with IVC is a safe and effective treatment for high‑risk NMIBC, with lower rates of recurrence, progression, tumor-specific death, PFS and RFS, and with minor and tolerable events. And the effectiveness of the IAC alone is parallel to the IVC alone. However, further quality studies are needed to evaluate its effectiveness due to low quality of evidence.
