# Supplementary material for: Application of intra-arterial chemotherapy in high-risk non-muscle invasive bladder cancer: a systematic review and meta-analysis
Source: PeerJ. 2021 Sep 28;9:e12248. doi: 10.7717/peerj.12248 (PMC8485834; doi:10.7717/peerj.12248)
Supplement: Supplemental Information 3 [file peerj-09-12248-s003.docx]

**ID**  **Search**

#1 bladder cancer [All fields]

#2 bladder cancers [All fields]

#3 bladder neoplasm [All fields]

#4 bladder neoplasms [All fields]

#5 bladder tumor [All fields]

#6 bladder tumors [All fields]

#7 urinary bladder neoplasms [MeSH terms]

#8 OR #1-7

#9 intra arterial [All fields]

#10 intra-arterial [All fields]

#11 intraarterial [All fields]

#12 OR #9-11

#13 intravesical [All fields]

#14 intra-vesical [All fields]

#15 bladder instillation [All fields]

#16 intravesical instillation [All fields]

#17 OR #13-16

#18 chemotherapy [All fields]

#19 drug therapy [MeSH terms]

#20 OR #18-19

#21 #8 AND #12 AND #20
